# The results of arthroscopic versus mini-open repair for rotator cuff tears at mid-term follow-up

**DOI:** 10.1186/1749-799X-2-24

**Published:** 2007-12-01

**Authors:** Albert W Pearsall, Khalid A Ibrahim, Sudhakar G Madanagopal

**Affiliations:** 1 Department of Orthopaedic Surgery, University of South Alabama, Mobile, Alabama, USA

## Abstract

**Background:**

To prospectively evaluate patients who underwent a "mini-open" repair versus a completely arthroscopic technique for small to large size rotator cuff tears.

**Methods:**

Fifty-two patients underwent "mini-open" or all arthroscopic repair of a full thickness tear of the rotator cuff. Patients who complained of shoulder pain and/or weakness and who had failed a minimum of 6 weeks of physical therapy and had at least one sub-acromial injection were surgical candidates. Pre and post-operative clinical evaluations included the following: 1) demographics; 2) Simple Shoulder Test (SST); 3) University of California, Los Angeles (UCLA) rating scale; 4) visual analog pain assessment (VAS); and 5) pre-op SF12 assessment. Descriptive analysis was performed for patient demographics and for all variables. Pre and post outcome scores, range of motion and pain scale were compared using paired t-tests. Analysis of variance (ANOVA) was used to evaluate any effect between dependent and independent variables. Significance was set at p is less than or equal to 0.05.

**Results:**

There were 31 females and 21 males. The average follow-up was 50.6 months (27 – 84 months). The average age was similar between the two groups [arthroscopic x = 55 years/mini-open x = 58 years, p = 0.7]. Twenty-seven patients underwent arthroscopic repair and 25 underwent repair with a mini-open incision. The average rotator cuff tear size was 3.1 cm (range: 1–5 centimeters). There was no significant difference in tear size between the two groups (arthroscopic group = 2.9 cm/mini-open group = 3.2 cm, p = 0.3). Overall, there was a significant improvement from pre-operative status in shoulder pain, shoulder function as measured on the Simple Shoulder test and UCLA Shoulder Form. Visual analog pain improved, on average, 4.4 points and the most recent Short Shoulder Form and UCLA scores were 8 and 26 respectively. Both active and passive glenohumeral joint range of motion improved significantly from pre-operatively.

**Conclusion:**

Based upon the number available, we found no statistical difference in outcome between the two groups, indicating that either procedure is efficacious in the treatment of small and medium size rotator cuff tears.

**Level of Evidence:**

Type III

## Background

Rotator cuff pathology is one of the most common conditions affecting the shoulder. Anatomic studies detailing rotator cuff tears in cadavers have noted a prevalence ranging from 17% to 72% [1-6]. Traditional treatment of full thickness tears of the rotator cuff has consisted of open surgical repair [7-9]. Reported satisfactory outcomes for open repair have ranged from 70% to 95% [9-22]. Although the effectiveness of open rotator cuff repair is well established, significant pain and morbidity can be associated with the procedure. A significant limitation to rehabilitation after open repair is pain associated with reattachment of the deltoid to the acromion. More recently, reports have described the evolution of rotator cuff repair to help minimize deltoid trauma and expedite post-operative rehabilitation. Good results have been reported with arthroscopically-assisted "mini-open" (< 3 cm incision) repair, as well as completely arthroscopic techniques [23-38]. Hata et al found that a mini-open repair caused less post-operative anterior deltoid atrophy, enabled earlier shoulder flexion, and resulted in improved UCLA Shoulder Scores when compared to a conventional open technique [39]. Kim et al retrospectively evaluated 76 patients who underwent arthroscopic versus mini-open salvage rotator cuff repair at an average of 39 months postoperatively. The authors noted no statistical difference in shoulder scores, pain and activity between the two techniques [40].

The senior author has evolved his technique of rotator cuff repair from a "mini-open" (< 3 cm) procedure to an all-arthroscopic procedure for tears up to 5 cm in diameter. The all-arthroscopic cases in the current report represent those after the senior author had mastered the learning curve for this difficult procedure. The purpose of the current study was to prospectively evaluate patients who underwent a "mini-open" repair versus a completely arthroscopic technique for small to large size rotator cuff tears.

## Methods

After obtaining institutional review board approval and written informed consent from the patients, 52 patients who underwent mini-open or all arthroscopic repair of a full thickness tear of the rotator cuff at our institution between 1999 and 2003 were evaluated in a prospective manner. Patients who complained of shoulder pain and/or weakness and who had failed a minimum of 6 weeks of physical therapy and had at least 1 subacromial injection by the senior author were surgical candidates. No patient presented with a history of an acute injury as the source of shoulder pain and all patients initially presented without an MRI. If the patient has been previously treated for a period of at least 3 months and continued to have symptoms, an MRI was ordered. All patients, regardless of age, had to have failed conservative treatment of a minimum of 6 weeks before surgical intervention was undertaken. All patients underwent a magnetic resonance imaging study of the affected shoulder without gadolium to assess for a rotator cuff tear. Not all patients in the study cohort had a MRI diagnosis of a rotator cuff tear prior to surgery. However, any patient who was diagnosed with a rotator cuff tear at the time of arthroscopy and met the inclusion criteria was included. Study inclusion criteria included the following: 1) a rotator cuff tear between 1 and 5 centimeters (measured at its greatest anterior-posterior width arthroscopically) treated with a mini-open (≤ 3 cm) incision or an all-arthroscopic technique; 2) a minimum follow-up of 24 months after surgery; and 3) completed pre-operative and post-operative evaluations. Patients who underwent concomitant distal clavicle excision, biceps tenolysis and glenohumeral debridement were included in the study analysis. Exclusion criteria included: 1) a massive rotator cuff tear (> 5 cm); 2) an acute tear repaired within 3 months after injury; 3) less than 24 month follow-up from surgery; 4) radiographic evidence of glenohumeral joint arthritis; and 5) any patient receiving workman's compensation. No patient performed predominately overhead activities for a living, although some patients did acknowledge that overhead activities were a small part of their occupation. No patient performed overhead sporting activities.

All pre and post-operative clinical and physical evaluations were performed by an independent examiner and included the following data: 1) demographics; 2) Simple Shoulder test (SST); 3) UCLA rating scale; 4) visual analog pain assessment (VAS); and 5) pre-op SF12 assessment. In addition, the following data was recorded during arthroscopic evaluation: 1) presence of long head biceps pathology; 2) humeral and/or glenoid full thickness articular cartilage defect (grade 0–2); and 3) rotator cuff tear size as measured at its greatest anterior-posterior diameter.

The UCLA Shoulder Score is a 35 point scale consisting of 10 points for pain, 10 points for function, and 5 points each for motion, strength, and patient satisfaction. A higher score indicates increased shoulder function. Although originally designed to assess outcome after shoulder arthroplasty, it is often used in the shoulder literature to assess results after rotator cuff repair [41,42].

The SST is a subjective questionnaire composed of 12 "yes" or "no" questions that assess shoulder pain and function. Although no formal scoring system is described for the SST, some researchers have reported results as total scores [43]. In the current study, a "yes" answer was allotted 1 point and a "no" answer given a score of 0. This resulted in a maximum possible score of 12, indicating greater shoulder function.

We used the SF-12 instead of the SF-36 since it has been reported there is significant correlation between the summary scores in rheumatoid arthritis and total knee patients [44,45]. Previous authors have described the use of the SF-36 to assess overall patient function and satisfaction after rotator cuff repair [41]. We are unaware of published reports evaluating the use of the SF-12 in comparison to other shoulder outcome scores to assess function after rotator cuff repair.

Active and passive glenohumeral motion was measured by 1 examiner (KAI), who was blinded to the patient's surgical procedure. Active forward flexion, glenohumeral abduction and internal rotation behind the back were measured with a goniometer recorded to the nearest 5 degrees. Maximum motion was recorded when full active abduction or flexion was achieved or at the point the patient began to demonstrate abnormal scapulothoracic motion to complete further shoulder elevation. Strength assessment was performed clinically and graded by the examiner as normal, weak or absent. Passive glenohumeral motion was measured in 4 directions: 1) isolated glenohumeral elevation; 2) humeral external rotation at 0 degrees of abduction; 3) humeral external rotation at 90 degrees of humeral abduction; and 4) humeral internal rotation at 90 degrees of humeral abduction. All motions were measured with the examiner using one hand to stabilize the scapula to insure that glenohumeral, not scapulothoracic, motion was being measured. Pre-operatively, each patient underwent physical evaluation to determine if he/she had acromioclavicular joint pain. These examination included palpation of the AC joint, the "cross-arm test" and the O'Brien test. If the patient had pain localized to the AC joint and had at least 1 of the remaining 2 tests positive, then it was determined that a distal claviculectomy would be performed at the time of surgery.

## Surgical Technique

### Mini-open

All procedures were performed with the patient in the beach-chair position. Patients were initially evaluated with glenohumeral arthroscopy to document intra-articular findings. Humeral head and glenoid articular surface integrity was evaluated. The long head of the biceps was evaluated. If the patients had pre-operative long head biceps symptoms and the tendon was frayed equal to or greater then 50% of its diameter, an arthroscopic biceps tenolysis was performed. For statistical purposes, articular cartilage findings were graded as 1 or 2. Normal articular cartilage or any defect not including exposed bone was classified as Grade I. Any exposed bone on the humerus and/or glenoid was classified as Grade 2. Any humeral head and/or glenoid articular defect that was Grade 1 was not addressed. All Grade 2 lesions were debrided at the time of surgery. No other procedures were performed to address humeral head and/or glenoid articular pathology. The articular footprint of the rotator cuff was inspected at its insertion on the humeral head. If any area appeared suspicious for a full thickness tear, a 2-0 prolene suture (Ethicon, Somerville, NJ) was passed from the skin though this portion of the rotator cuff into the joint. The bursal side of the tendon at the site of the suture was subsequently inspected during the subacromial evaluation.

Patients undergoing mini-open repair underwent an arthroscopic subacromial inspection and documentation of the rotator cuff location and size. Rotator cuff tear size was measured with an arthroscopic probe at the point of greatest anterior-posterior diameter. Through an antero-lateral portal, an arthroscopic subacromial decompression of 5 mm-8 mm was performed from the antero-lateral acromion to the junction of the acromio-clavicular joint in 92% of patients. The remaining patients were felt to have adequate subacromial space that did not necessitate a subacromial decompression. If a distal clavulectomy was not performed, the acromio-clavicular joint ligaments were not disrupted and "co-planing" of the undersurface of the distal clavicle was not done. Eighty-four percent of distal clavulectomies were performed arthroscopically, with 16% performed open. In all instances, 8 mm-10 mm of distal clavicle was resected. A subacromial bursectomy was performed and the rotator cuff debrided. The antero-lateral portal was then extended 3 cm for a mini-open repair. After splitting the deltoid, all rotator cuff tears were re-measured at the greatest antero-postero diameter of the tear to insure accuracy. Using a burr, the surface of the greater tuberosity was superficially abraded. This area began at the articular footprint and extended to the greater tuberosity, approximately 10 millimeters. The anterior posterior dimensions of the abrasion were based upon the size of the tear. Between 1–3 bioabsorbable suture anchors (Arthrex, Naples, Florida) were placed. In the medial-lateral dimension, the anchors were placed midway between the articular surface and greater tuberosity. Depending upon the anterior-posterior dimensions of the tear, an attempt was made to arrange the anchors to cover the footprint with the repaired tendon. A free needle was used to secure the sutures through the tendon with a simple stitch and all knots were tied with four alternating half hitches. The arm was internally and externally rotated to inspect the repair and the deltoid and skin closed in an interrupted fashion.

All patients remained in a sling for 6 weeks and were allowed passive motion under the direction of a physical therapist after the first week. After 6 weeks, progressive active motion and strengthening was instituted for a total of 3 months.

### Arthroscopic

Mastery of the "all-arthroscopic" technique of rotator cuff repair has a steep learning curve. The senior author (AWP) did not want to bias the results of the current study due to technical errors that were a direct result of this learning curve. Consequently, the first 20 arthroscopic rotator cuff repairs that met the study inclusion criteria were not included in the current study. Only patients later than this group who met the study inclusion criteria were considered for inclusion in the current study.

The glenohumeral joint and initial subacromial arthroscopic evaluation for the arthroscopic repair was identical to that performed for a mini-open procedure. After sizing the rotator cuff tear and mobilizing the tendon, an arthroscopic greater tuberosity abrasion was performed as previously described and 1–3 suture anchors (Arthrex, Naples, Florida) placed. Care was taken to reproduce the anatomic footprint with repair of the rotator cuff. Using an arthroscopic suture passer (Mitek, Westwood, MA), anchor sutures were passed through the tendon in a simple stitch manner. All knots were tied with four alternating half hitches. The arm was internally and externally rotated to inspect the repair and the portals closed.

The postoperative regimen for the arthroscopic repair was identical to that for the mini-open repair.

### Analysis of the Data and Statistics

Descriptive analysis was performed for patient demographics and for all variables. Pre and post outcome scores, range of motion and pain scale were compared using paired t-tests. ANOVA was used to evaluate any effect between dependent and independent variables. Correlation analysis was performed between outcome scores as well as between independent variables and outcome measures. Significance was set at p ≤ 0.05.

## Results

A total of 54 patients met the study inclusion criteria. Fifty-two underwent physical examination and completed the follow-up questionnaires. This cohort constituted the study group (93% follow-up). There were 31 females and 21 males. The average follow-up was 50.6 months (27 – 84 months). The average age was similar between the two groups [arthroscopic x = 55 years [range: 38–78]/mini-open x = 58 years [range: 41–76] p = 0.7]. Twenty-seven patients underwent arthroscopic repair and 25 underwent repair with a mini-open incision. The average duration of symptoms was 5.7 months (range: 3–16 months). The average rotator cuff tear size was 3.1 cm (range: 1–5 centimeters). There was no significant difference in tear size between the two groups (arthroscopic = 2.9 cm/mini-open = 3.2 cm, p = 0.3). Pre-operative magnetic resonance imaging detected a full or partial thickness tear in only 58% of patients, demonstrated no tear in 8% and was inconclusive in 34%. Twenty-two percent of patients had diabetes mellitus and 22% also had a history of smoking. Based upon arthroscopic findings, 56% of patients had biceps tendon pathology, and 25% had glenoid and/or humeral arthritis (Table 1). All biceps pathology was classified as fraying of the tendon with no instances of SLAP tears.

**Table 1:** Demographic variables between patients undergoing arthroscopic versus a mini-open technique.

**Category**		**Mini-open**	**Arthroscopic**	**P value**
**Study Group**	N = 52	25	27	
**Average age (years)**	56	55	58	*p *= 0.13
**Sex male/female**	21/31	10/17	11/14	*p *= 0.7
**Smoking**	21%	30%	12%	*p *= 0.17
**Diabetes**	21%	19%	24%	*p *= 0.7
**Biceps pathology**	55%	48%	64%	*p *= 0.27
**Humeral osteoarthritis**	15%	15%	16%	*p *= 1.0
**Glenoid osteoarthritis**	10%	11%	8%	*p *= 1.0
**Tear size (centimeters)**	3.1	2.9	3.2	*p *= 0.3
**Number of anchors**	--	2.1	2.0	*p *= 0.29
**Distal clavicle excision**	25	14	11	--

Overall, there was a significant improvement at the most recent follow-up from pre-operative status in shoulder pain, shoulder function as measured on the Simple Shoulder test and UCLA Shoulder Form. On average, visual analog pain improved 4.4 points and the most recent Short Shoulder Form and UCLA scores were 8 and 26 respectively. Both active and passive glenohumeral joint range of motion also improved significantly from pre-operatively (Table 2).

**Table 2:** Pre-operative and follow-up values for shoulder pain, active and passive glenohumeral motion.

**Category**	**Pre-Operative Value**	**Post-Operative Value**	**P value**
**Pain**	7.8	**3.4**	p < 0.0001*
**Short Shoulder Form**	2.9	7.9	p < 0.0001*
**SF-12**	31.8	32	p = 0.8
**UCLA Score**	14	31	p < 0.0001*
**Active forward flexion (degrees)**	125	152	P = 0.01*
**Active abduction (degrees)**	121	139	P = 0.07
**Glenohumeral elevation (degrees)**	80	87	P = 0.01*
**External rotation @ 0 (degrees)**	48	59	P = 0.03*
**External rotation @ 90 (degrees)**	53	70	P = 0.001*
**Internal rotation @ 90 (degrees)**	46	56	P = 0.14

* = significant

In order to compare the results of arthroscopic and mini-open rotator cuff repair techniques, these two groups were analyzed separately. When post-operative improvement was compared between groups for the UCLA Score, Simple shoulder test, VAS for the shoulder, and active and passive glenohumeral motion, no significant difference was noted (Table 3). Power was calculated to be 0.07. In order to confirm the hypothesis that both mini-open and arthroscopic techniques have similar results with a power value of 0.8, we calculated that 511 patients in each group would be required, assuming the current mean scores and standard deviations.

**Table 3:** Comparison of outcome improvement between arthroscopic and mini-open rotator cuff repair patients.

**Outcome Measure**	**Arthroscopic**	**Mini-open**	**P value**
**Study Group (N = 52)**	N = 27	N = 25	p = 0.13
**UCLA Score**	24	27	p = 0.34
**Short Shoulder Test Improvement**	5.1	4.7	p = 0.66
**VAS Pain Improvement**	3.9	4.8	p = 0.29
**Active forward flexion improvement (degrees)**	35	18	p = 0.16
**Active abduction improvement (degrees)**	21	14	P = 0.18
**Glenohumeral elevation improvement (degrees)**	8.3	7.0	p = 0.7
**External rotation @ 0 improvement (degrees)**	11	12	p = 0.7
**External rotation @ 90 improvement (degrees)**	19	16	p = 0.7
**Internal rotation @ 90 improvement (degrees)**	8	11	p = 0.7

A correlation analysis was performed between all demographic variables and outcome measures for the entire group (N = 52). When all variables were analyzed, an inverse correlation was found between smoking and improvement on the Short Shoulder Form (p = 0.05). This indicated that patients who smoked had less improvement on the SSF than those who did not smoke. A strong correlation (p = 0.03) was noted between tear size and VAS improvement, suggesting that patients with larger tears did not have as much pain relief after repair. The presence of glenoid or humeral osteoarthritis did affect the UCLA score improvement significantly (p = 0.05). No correlation was found between age, sex, presence of diabetes, biceps pathology, concomitant distal clavicle excision and improvement in any of the outcome variable or glenohumeral range of motion.

## Discussion

The gold standard for treatment of symptomatic full thickness rotator cuff tears has historically been open rotator cuff repair as pioneered by Codman [46]. Klepps et al and others have documented the validity and reproducibility of this procedure [13,15,47-50]. Despite good results reported with open rotator cuff repair, significant morbidity and prolonged rehabilitation have been associated with the requisite deltoid take-down and repair [9,48,51-53]. In response to reports of prolonged pain and rehabilitation after open rotator cuff repair, the arthroscopically assisted "mini-open" or "portal-extension" technique was popularized [23-26,54-61]. In an effort to further decrease post-operative pain and rehabilitation time, Johnson described the first completely arthroscopic rotator cuff repair [62]. Since the introduction of the all-arthroscopic rotator cuff repair technique, there has been considerable debate over the benefits of this procedure versus the "mini-open" technique. Several reports have documented good results after arthroscopic repair [33,35,37,38,40,62-64]. Numerous reports have also touted the arthroscopically-assisted "mini-open" procedure (< 3 cm) for small and medium sized tears of the rotator cuff [23-26,28,54-57,59,60].

The current study evaluated functional outcome in similar patient groups undergoing arthroscopically-assisted or completely arthroscopic rotator cuff repair. With the numbers available, there was no statistical difference between the two groups for any independent variable. (Table 1). When data at the most recent follow-up was compared to pre-operatively for the whole group, there was a statistical improvement in 7 out of 9 clinical parameters. Although active internal rotation was improved compared to pre-operatively, the improvement did not meet statistical significance. Finally SF-12 scores were essentially unchanged from pre-operatively. Since the SF-12 measures well being, in addition to physical parameters, several parameters not-related to the patients' shoulder may have contributed to this lack of improvement [41]. For both groups, the overall improvement observed in pain and function is comparable to reports by other authors [41].

The amount of biceps pathology noted in our study was over 50%. We attributed this relatively high prevalence of biceps abnormalities to the strict criteria used in our evaluation. Any fraying of the long head of the biceps was considered abnormal. The strict criteria followed may have over-classified biceps abnormalities that did not correlate clinically.

In order to better analyze outcome, ANOVA was performed to analyze the outcome improvement between the 2 groups for the 9 measures used in the study. We found no statistical difference in improvement between the 2 groups for any variable. With the numbers available, we found no statistical difference in shoulder range of motion, pain, or functional outcome between an arthroscopically-assisted or completely arthroscopic technique.

Our analysis using the SF-36 outcome measures demonstrated no significant difference between pre and post operative scores, despite having significant improvement in SST, UCLA and Constant & Murley scores. This is in agreement with Gartsman et al who have used UCLA, Constant & Murley and SF-36 forms to evaluate patients after rotator cuff repair [41,65].

There are several weaknesses to the current study. The data is limited to one surgeon and may not necessarily be applied to all surgeons who perform rotator cuff repairs with varying skill levels. The numbers in the current study are relatively small. With the numbers available, we did not achieve statistical power (power = 0.07). In order to statistically confirm that both mini-open and arthroscopic techniques have similar results with a power value of 0.8 and alpha value of 0.05, we would require 511 patients in each group assuming the current mean scores and standard deviation. Although the authors standardized the post-operative physical therapy regimen, we did not have the same therapist for all patients. This potential variability in post-operative treatment may have influenced the outcome in some patients.

MRI accuracy in the current study was 58%, with 42% of full thickness tears missed. Although the increased number of MRI misdiagnosed complete rotator cuff tears is a cause for concern, we do not believe that this weakness had any bearing on the indications, surgical intervention, nor outcome of the study cohort. Certainly, all patients who underwent surgical intervention failed at least 3 months of conservative treatment, regardless of whether the pre-operative MRI demonstrated a full thickness tear. Arguably, if post-operative magnetic resonance imaging were to be used to evaluate cuff integrity, the current imaging techniques at our institution would be called into question. However, when using the clinical criteria and post-operative measures currently used, we do not believe this weakness in the current study confounded any outcome variable.

Finally, we did not perform magnetic resonance imaging or ultrasonography on all patients at the most recent follow-up. Several authors have described the lack of integrity of rotator cuff repairs when analyzed with these modalities [47,66]. Despite these reports, the lack of rotator cuff integrity may not correlate with clinical outcome [47]. Currently the authors obtain magnetic resonance imaging of all patients' operated shoulders at yearly intervals. However, the current data indicates no significant difference in clinical outcome between the 2 groups. Such imaging data may be more pertinent in evaluating the technical aspects of repair in the 2 groups or as a component of outcome analysis at longer term follow-up.

## Conclusion

In conclusion, the current study evaluated the clinical outcome of patients undergoing an arthroscopically-assisted or completely arthroscopic technique for repair of a small or medium rotator cuff tear. Based upon the number available, we found no statistical difference in outcome between the two groups, indicating that either procedure is efficacious in the treatment of small and medium size rotator cuff tears.

## Competing interests

The author(s) declare that they have no competing interests.

## Authors' contributions

AWP – Wrote manuscript/data analysis.

KAI – Collected data/data analysis.

SGM – Data analysis/assisted with manuscript.

All authors read and approved the final manuscript.
